# Network Analysis and Nomogram in the Novel Classification and Prognosis Prediction of Advanced Schistosomiasis Japonica

**DOI:** 10.4269/ajtmh.22-0267

**Published:** 2023-01-23

**Authors:** Xue-Fei Liu, Ying Li, Shuai Ju, Yan-Li Zhou, Jin-Wei Qiang

**Affiliations:** ^1^ Department of Radiology, Jinshan Hospital, Fudan University, 201508, Shanghai, China;; ^2^ Department of Interventional Radiology, Jinshan Hospital, Fudan University, 201508, Shanghai, China;; ^3^ Department of Nuclear Medicine, Jinshan Hospital, Fudan University, 201508, Shanghai, China

## Abstract

Clinical classification of advanced schistosomiasis japonica is important for treatment options and prognosis prediction. Network analysis was used to solve the problem of complexity and co-occurrence complications in classification of advanced schistosomiasis. A total of 4,125 retrospective patients were enrolled and divided randomly into a training cohort (*n* = 2,888) and a validation cohort (*n* = 1,237). Network analysis was used to cluster the isolated complications of advanced schistosomiasis. The accuracy of the network was evaluated. Nomograms based on the clustered complications were built to predict 1- to 5-year survival rates in advanced schistosomiasis. The predictive performance of the nomogram was also evaluated and validated. Fifteen isolated complications were identified: metabolic syndromes, minimal hepatic encephalopathy, hepatic encephalopathy, chronic obstructive pulmonary disease, pulmonary hypertension, respiratory failure, right heart failure, gastroesophageal variceal bleeding, gastrointestinal ulcer bleeding, splenomegaly, fibrosis, chronic kidney disease, ascites, colorectal polyp, and colorectal cancer. Through network analysis, three major clustered complications were achieved—namely, schistosomal abnormal metabolic syndromes (related to chronic metabolic abnormalities), schistosomal abnormal hemodynamics syndromes (related to severe portal hypertension and portosystemic shunting), and schistosomal inflammatory granulomatous syndromes (related to granulomatous inflammation). The nomograms showed a good performance in prognosis prediction of advanced schistosomiasis. The novel classification-based nomogram was useful in predicting the survival rate in advanced schistosomiasis japonica.

## INTRODUCTION

Schistosomiasis is a neglected tropical disease afflicting more than 250 million people worldwide.^1^ The disease is estimated to cause disability-adjusted life-year losses amounting to 3.5 million in 2105.^2^ In China, hepatointestinal schistosomiasis is caused by *Schistoma japonicum*.^3^ In general, the natural course of hepatointestinal schistosomiasis japonica is classified as acute schistosomiasis, chronic schistosomiasis, and advanced schistosomiasis.^4^^,^^5^

The pathogenic basis of advanced schistosomiasis japonica is based on the immune response of the host to the eggs of a schistosome, which results in granulomas and extensive fibrosis in the liver and intestinal wall (especially the wall of the colon).^6^ The diagnostic criteria of schistosomiasis japonica was proposed previously^7^ by medical scientists and experts as follows: 1) a long or repeated history of schistosome cercarial-infested water contact or a confirmed schistosomiasis treatment history; 2) hepatic fibrosis and portal hypertension syndrome or serious growth and development hindrance, such as dwarfism or significant intestinal wall granulomas; and 3) schistosome eggs or miracidia found by stool examination, the eggs found by rectum wall biopsy, or confirmed positive results of serological examinations. In 2012, Deng et al.^4^ proposed a classification of advanced schistosomiasis japonica based on syndromes related to liver fibrosis and portal hypertension. Eight subtypes—huge splenomegaly, ascites, colon proliferative, dwarfism, universal, bleeding, hepatic coma, and miscellaneous—were proposed.

Clinical classification has a significant impact on the treatment and prognosis of advanced schistosomiasis, which poses a great challenge and impact on human health and the economy.^8^ However, with the course of schistosomiasis prolongation, diagnostic technology improvement, and treatment methods renewal, the current classification is outmoded and insufficient in routine clinical practice.^9^ For example, dwarfism, is very rare in advanced schistosomiasis.^4^ Some common complications are not included in the existing classification, such as pulmonary hypertension, chronic kidney disease, minimal hepatic encephalopathy, and metabolic syndrome (including hypertension, diabetes, and hyperlipidemia).^10^^,^^11^ Furthermore, the complications are usually co-occurring rather than presenting as isolated complications.^4^ Thus, a more comprehensive classification is required to meet the clinical needs for therapy choice and prognosis prediction in advanced schistosomiasis.

As a result of the complexity and particularity of advanced schistosomiasis, the complications should be analyzed with rigorous theoretical networks rather than as coupled interactions in isolation. Network analysis—an increasingly popular approach—has been used to detect the distances (homogeneity/heterogeneity) between clinical symptoms for etiological underpinnings in psychiatric disorders.^12^ Networks allow the representation of complex phenomena in terms of a set of elements that interacts with each other. Networks include two basic components: the nodes, which represent the elements of a system; and the edges, which connect nodes and represent their pairwise interactions.^13^ Network analysis may provide useful approaches to identify subgroups of patients with distinct symptom profiles.

Currently, no clinical classification provides prediction of the prognosis in advanced schistosomiasis with regard to the complications as co-occurring networks. In attempt to solve this problem, network analysis was used to explore a more practical and scientific, standardized clinical classification in advanced schistosomiasis. The prediction ability of this novel classification on the 5-year overall survival rate is also reported.

## MATERIALS AND METHODS

This retrospective study was approved by the Ethics Committee of Jinshan Hospital, Fudan University (no. JIEC 2021-S55), and informed consent was waived. Patient confidentiality was protected by ensuring the data addressed was anonymous, with personal information appropriately de-identified.

### Study population.

**Figure 1.**
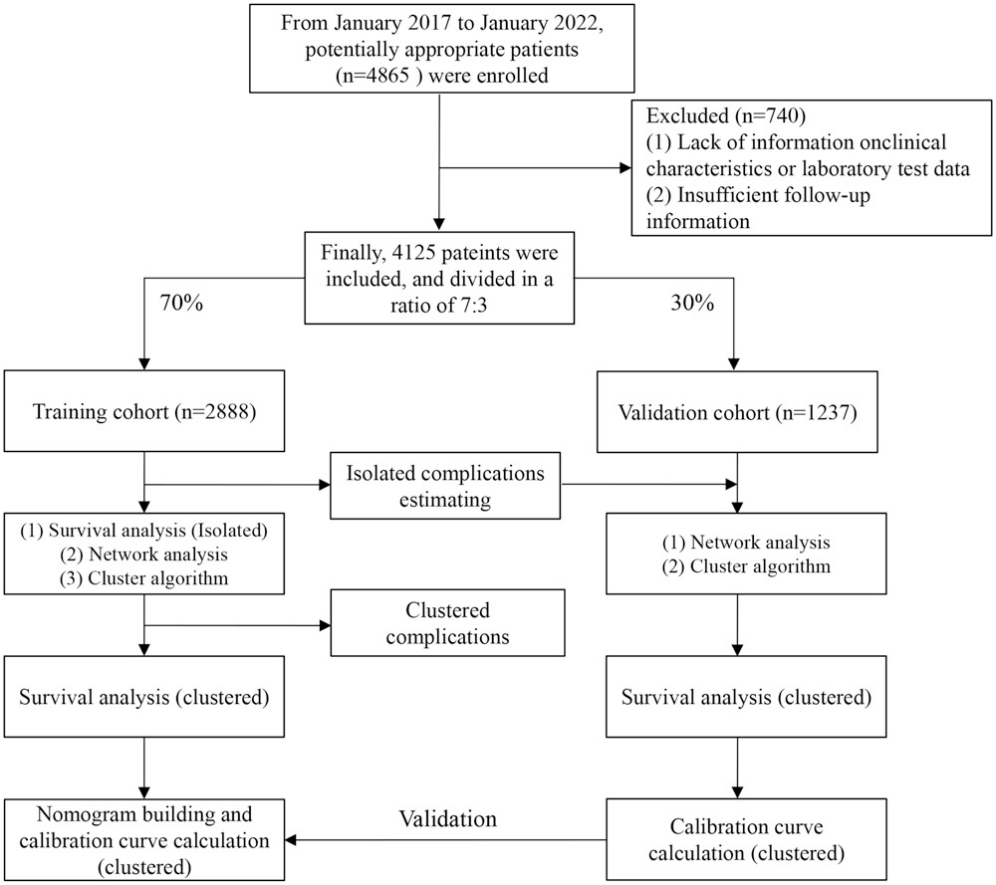
The work flow of our study.

From January 2017 to January 2022, 4,865 first-visit inpatients with advanced schistosomiasis japonica were enrolled retrospectively.^7^ The electronic medical records of the potentially appropriate patients were reviewed. According to the total number of the collected cases, we conducted a careful screening. The inclusion criteria were as follows: 1) portal hypertension and portosystemic shunting by imaging showing widened portal vein and shunting vessels; 2) involvement of advanced schistosomiasis complications including ascites, splenomegaly, portal hypertension, and gastroesophageal variceal bleeding, or with granulomatous lesions of the colon and rectum, or severe growth retardation; and 3) patients hospitalized to receive surgical or medical treatment of schistosomiasis complications. The exclusion criteria were as follows: 1) lack of information on clinical characteristics or laboratory tests and 2) insufficient follow-up information on survival outcome. After applying these criteria, 4,125 patients were included in the study. The patients were divided into a training cohort (*n* = 2,888) and a validation cohort (*n* = 1,237) at a ratio of 7:3. The work flow is shown in Figure 1.

From the electronic medical records, 15 commonly isolated complications were identified preliminarily: metabolic syndromes, minimal hepatic encephalopathy, hepatic encephalopathy, chronic obstructive pulmonary disease (COPD), pulmonary hypertension, respiratory failure, right heart failure, gastroesophageal variceal bleeding, gastrointestinal ulcer bleeding, splenomegaly, fibrosis, chronic kidney disease (CKD), ascites, colorectal polyp, and colorectal cancer. Because there were few cases of dwarfism, we did not include this in further analyses. The isolated complications were treated as dichotomous values. Values were marked as positive if any of the isolated complications existed; otherwise, they were marked as negative.

### Network analysis and cluster of the isolated complications.

A network analysis was performed by taking each isolated complication as the “node” of the network, and the correlation coefficient (by Spearman’s correlation) between (connected) the nodes as the “edge.” The relationship between the nodes was then calculated and analyzed using an IsingFit model for binary variables in which the edges were akin to partial correlations.^14^

Node centrality (quantifying the importance of a node in the network) was calculated to describe the network, including node strength (quantifying how strongly a node connected directly to another), closeness (quantifying how strongly a node connected indirectly to another), and betweenness (quantifying the importance of a node in the average path between two other nodes). Strength was the main evaluation indicator of node centrality. The correlation–stability coefficient (0-0.24, poor; 0.25–0.49, fine; 0.50–0.75, preferable; 0.76-1, good) by case-dropping subset bootstrap was used to test node centrality stability. The accuracy of edge weight (quantifying how strong/weak and positive/negative the relationships were between the nodes) was evaluated. Clusters of local strongly interconnected nodes were identified using the Spinglass algorithm.^12^ Node centrality, edge-weight accuracy of the network, and the Spinglass algorithm were also determined/performed for the validation cohort.

### Survival analysis in clustered complications, nomogram building, and validation.

The survival curves of each cluster (clustered complications) were plotted. We established a nomogram based on the clustered complications from the network analysis to predict the overall 5-year survival rate in advanced schistosomiasis. A certain cluster (stored as a binary variable) was labeled as positive if any isolated complication within this cluster presented. All the clusters, along with the age of the training cohort, were integrated into a nomogram to predict the survival rate of individual patients with advanced schistosomiasis. Calibration curves were used to evaluate the prediction ability of the nomogram, which was also validated.

### Statistical analysis.

Statistical analysis was performed using R software (version 4.0.5; R Foundation for Statistical Computing, Vienna, Austria). The gganatogram package was used for anatogram plotting; the qgraph, bootnet, and igraph packages were used for network analysis and the Spinglass algorithm; the rms package was used for nomogram and calibration curve plotting; and the survival and survminer packages were used for survival analysis. The association between the isolated complications was assessed using Spearman’s correlation. Survival analysis was assessed by Kaplan–Meier curves via log-rank tests. *P* < 0.05 was considered statistically significant.

## RESULTS

### Study population.

In the included patients with advanced schistosomiasis (mean age ± SD, 76 ± 7.7 years; range, 32–100 years), 1,821 were women and 2,304 were men. Baseline clinical characteristics of the training cohort and the validation cohort are presented in Table 1.

**Table 1:** Baseline characteristics of the training cohort and the validation cohort

Characteristic	Training cohort (*n* = 2,888 , *n *(%)*	Validation cohort (*n* = 1,237) , *n *(%)*	*P* value
Gender			
Female	1,285 (44.5)	536 (43.3)	0.512
Male	1,603 (55.5)	701 (56.7)	
Age, years; mean (SD)	76.3 (7.70)	76.3 (7.80)	0.982
Metabolic syndromes			
−	1,049 (36.3)	453 (36.6)	0.883
+	1,839 (63.7)	784 (63.4)	
Hepatic encephalopathy			
−	2,791 (96.6)	1,203 (97.3)	0.354
+	97 (3.4)	34 (2.7)	
Minimal hepatic encephalopathy			
−	2,618 (90.7)	1,107 (89.5)	0.273
+	270 (9.3)	130 (10.5)	
Chronic obstructive pulmonary disease			
−	2,596 (89.9)	1,119 (90.5)	0.613
+	292 (10.1)	118 (9.5)	
Respiratory failure			
−	2,698 (93.4)	1,132 (91.5)	0.034
+	190 (6.6)	105 (8.5)	
Pulmonary hypertension			
−	2,543 (88.1)	1,097 (88.7)	0.602
+	345 (11.9)	140 (11.3)	
Right heart failure			
−	2,617 (90.6)	1,113 (90.0)	0.56
+	271 (9.4)	124 (10.0)	
Gastroesophageal variceal bleeding			
−	2,767 (95.8)	1,190 (96.2)	0.621
+	121 (4.2)	47 (3.8)	
Gastrointestinal ulcer bleeding			
−	2,319 (80.3)	1,009 (81.6)	0.366
+	569 (19.7)	228 (18.4)	
Splenomegaly			
−	2,677 (92.7)	1,163 (94.0)	0.142
+	211 (7.3)	74 (6.0)	
Fibrosis			
−	906 (31.4)	400 (32.3)	0.566
+	1,982 (68.6)	837 (67.7)	
Chronic kidney disease			
−	2,383 (82.5)	1,019 (82.4)	0.951
+	505 (17.5)	218 (17.6)	
Ascites			
−	2,552 (88.4)	1,102 (89.1)	0.539
+	336 (11.6)	135 (10.9)	
Colorectal polyp			
−	2,671 (92.5)	1,142 (92.3)	0.904
+	217 (7.5)	95 (7.7)	
Colorectal cancer			
−	2,788 (96.5)	1,209 (97.7)	0.053
+	100 (3.5)	28 (2.3,%)	

* Except where indicated.

### Preliminary estimating and survival analysis in isolated complications.

**Figure 2.**
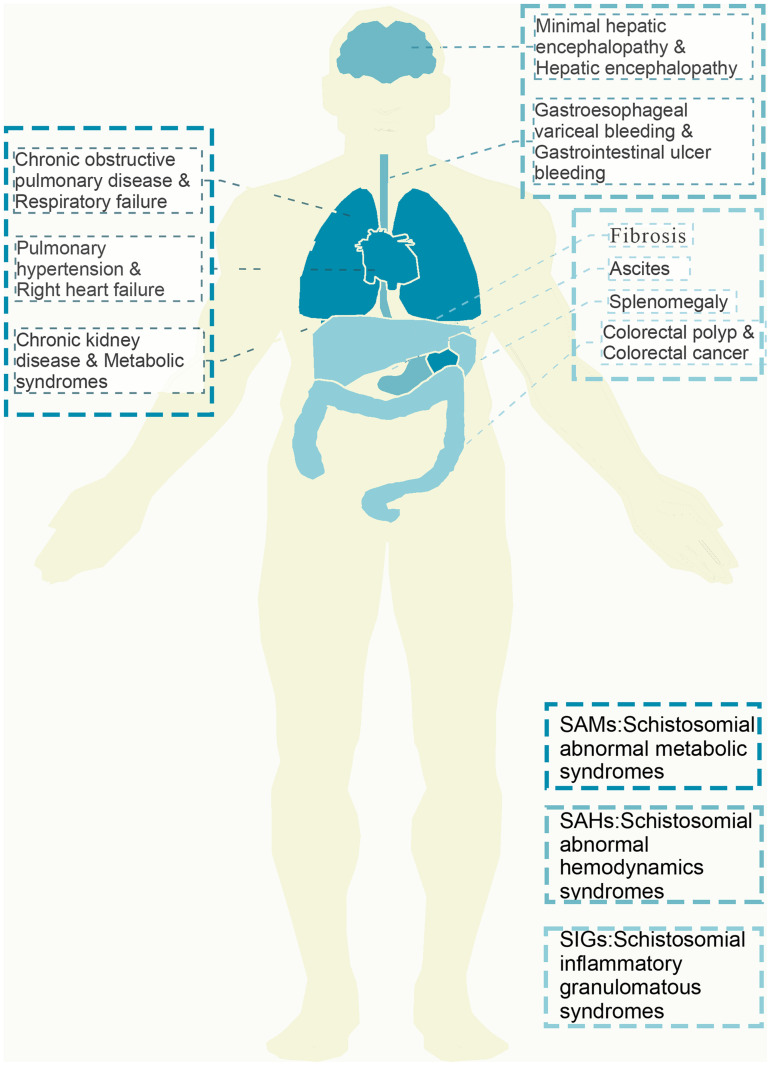
The 15 isolated complications of advanced schistosomiasis.

The prevalence of the 15 isolated complications were as follows: metabolic syndrome, 63.6%; minimal hepatic encephalopathy, 9.7%; hepatic encephalopathy, 3.2%; COPD, 9.9%; pulmonary hypertension, 11.8%; respiratory failure, 7.2%; right heart failure, 9.6%; gastroesophageal variceal bleeding, 4.1%; gastrointestinal ulcer bleeding, 19.3%; splenomegaly, 6.9%; fibrosis, 68.3%; CKD, 17.5%; ascites, 11.4%; colorectal polyp, 7.6%; and colorectal cancer, 3.1%. The 15 isolated complications are shown in Figure 2.

The survival rates of the patients with and without each isolated complication were different in hepatic encephalopathy (*P* < 0.001), minimal hepatic encephalopathy (*P* < 0.001), COPD (*P* = 0.014), respiratory failure (*P* < 0.001), pulmonary hypertension (*P* < 0.001), right heart failure (*P* < 0.001), gastroesophageal variceal bleeding (*P* < 0.001), splenomegaly (*P* < 0.001), CKD (*P* < 0.001), ascites (*P* < 0.001), and colorectal polyp (*P* < 0.001). No differences were seen in metabolic syndrome (*P* = 0.250), gastrointestinal ulcer bleeding (*P* = 0.750), fibrosis (*P* = 0.210), and colorectal cancer (*P* = 0.790) (Supplemental Figure S1).

### Network analysis and cluster of the isolated complications.

**Figure 3.**
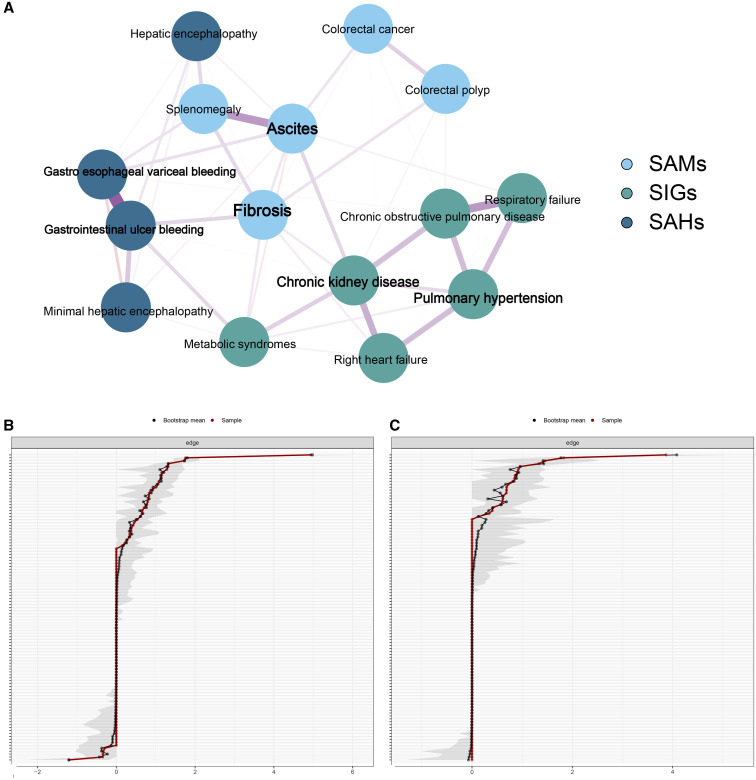
Estimated network structure based on the training cohort using the IsingFit model (the Fruchterman–Reingold algorithm was used for node placement). (**A**) The Spinglass algorithm clusters the robust edges network in three major clusters. Bootstrapped confidence intervals of estimated edge weights for the network in training cohort (**B**) and the validation cohort (**C**) are shown. The red line indicates the sample values. The gray shading indicates the bootstrapped CIs. SAHs = schistosomal abnormal hemodynamics syndromes; SAMs = schistosomal abnormal metabolic syndromes; SIGs = schistosomal inflammatory granulomatous syndromes.

Through network analysis, three major clusters were noted. We named the first cluster as schistosomal abnormal metabolic syndromes (SAMs), and they included metabolic syndrome, CKD, pulmonary hypertension, right heart failure, COPD, and respiratory failure, which were related to chronic metabolic abnormalities. We named the second cluster schistosomal abnormal hemodynamics syndromes (SAHs), and they included minimal hepatic encephalopathy, hepatic encephalopathy, gastroesophageal variceal bleeding, and gastrointestinal ulcer bleeding, which were related to severe portal hypertension and portosystemic shunting. We designated the third cluster schistosomal inflammatory granulomatous syndromes (SIGs), and they included fibrosis, ascites, splenomegaly, colorectal polyp, and colorectal cancer, which were related to fibrosis and proliferation of the portal system, and colorectal mucosa secondary to granulomatous inflammation (Figure 3). The network structure of the validation cohort is shown in Supplemental Figure S2.

Node centrality is shown in Supplemental Table S1. CKD and pulmonary hypertension were the most important nodes in the sub-net of SAMs, gastroesophageal variceal bleeding and gastrointestinal ulcer bleeding were the most important nodes in the sub-net of SAHs, and fibrosis and ascites were the most important nodes in the sub-net of SIGs. The correlation–stability coefficients of the strength, closeness, and betweenness were 0.595, 0.283, and 0.361 in the training cohort; and 0.594, 0.205, and 0.005 in the validation cohort respectively. Results indicated a preferable node centrality stability. A good edge-weight accuracy is shown in Figure 3.

### **Survival analysis in clustered complications and nomogram building, and validation**.

**Figure 4.**
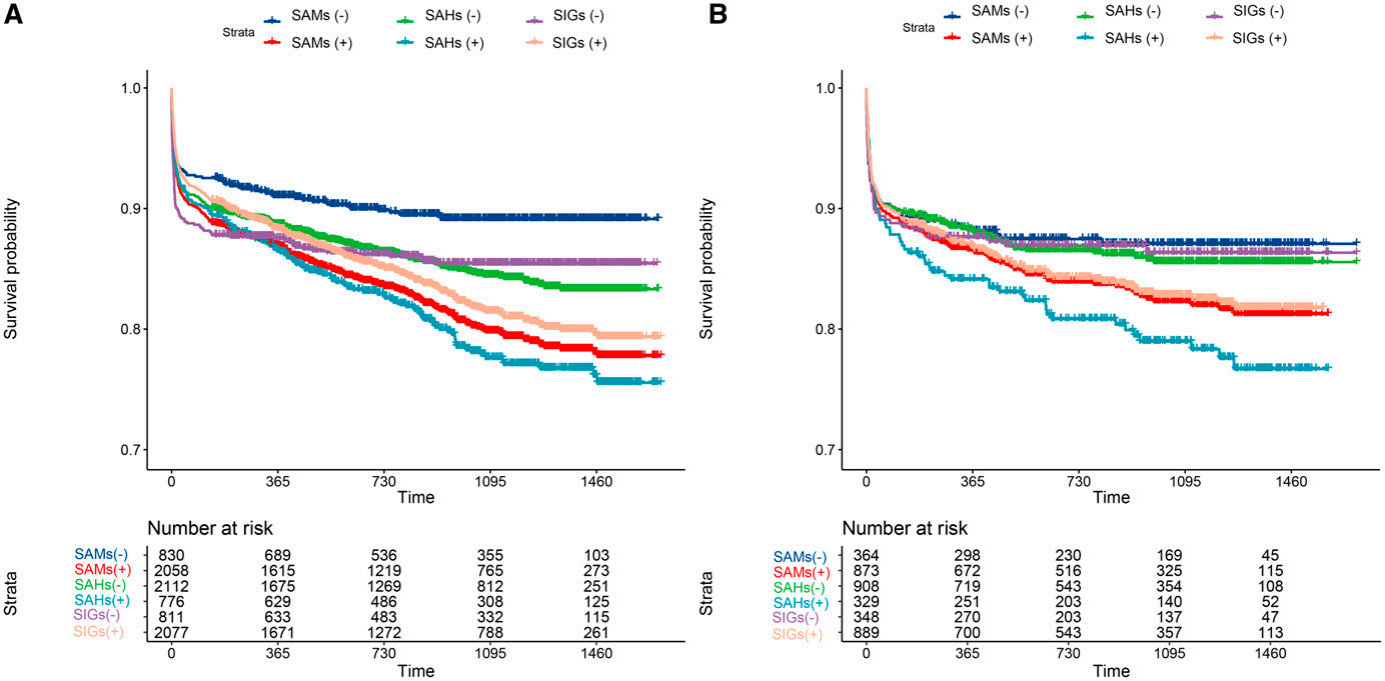
The Kaplan–Meier analysis of three major clustered complications of advanced schistosomiasis in the training cohort (**A**) and the validation cohort (**B**). SAHs = schistosomal abnormal hemodynamics syndromes; SAMs = schistosomal abnormal metabolic syndromes; SIGs = schistosomal inflammatory granulomatous syndromes.

**Figure 5.**
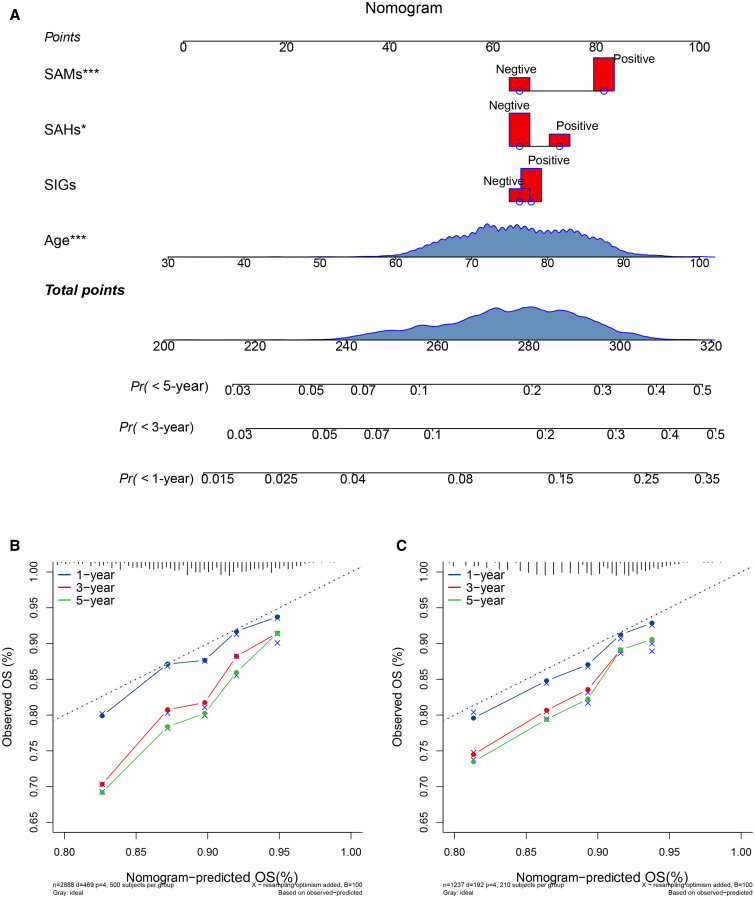
The nomogram and calibration curves. (**A**) The nomogram is constructed by integrating the three major clustered complications of advanced schistosomiasis with patient age to predict the 1- to 5-year survival probability. The calibration curves of the nomogram in the training cohort (**B**) and the validation cohort (**C**) are shown. Based on our nomogram, for example, a 70-year-old patient (60 points) with any positive of schistosomal abnormal metabolic syndrome ([SAM] 80 points), any positive schistosomal abnormal hemodynamics syndrome ([SAH] 75 points), and any positive of schistosomal inflammatory granulomatous syndrome ([SIGs] 70 points) has a total of 285 points, which corresponds to a probability of approximately 25% of having a survival rate less than 5 years (20% for 3 years and 15% for 1 year). OS = overall survival; *Pr* = probability. SAHs = schistosomal abnormal hemodynamics syndromes; SAMs = schistosomal abnormal metabolic syndromes; SIGs = schistosomal inflammatory granulomatous syndromes.

Survival analysis indicated that the overall 5-year survival rate was low in SAHs, median in SAMs, and high in SIGs (Figure 4). Figure 5 illustrates the predictive nomogram for the 1-, 3-, and 5-year survival rate for advanced schistosomiasis. Each variable (including age, SAMs, SAHs, and SIGs) was projected upward to the score (points) of the ruler. By adding the scores of each selected variable, a total score (total points) for each patient was determined to calculate the probability of individual survival. The higher the score, the worse the survival rate. According to the nomogram, we could predict the prognosis based on the different characteristics of each patient. The performance (area under the receiver–operating characteristic curve) of the nomogram in predicting the survival of patients with advanced schistosomiasis japonica was 0.72 (95% CI, 0.70–0.74), 0.60 (95% CI, 0.59–0.62), and 0.63 (95% CI, 0.58–0.68) for 1, 3, and 5 years in the training cohort, and 0.71 (95% CI, 0.69–0.73), 0.61 (95% CI, 0.59–0.63), and 0.63 (95% CI, 0.59–0.67) for 1, 3, and 5 years in the validation cohort. Calibration curves (Figure 5) showed that the nomogram had good prediction performance for 1-year survival rates both in the training and validation cohorts, but the nomogram underestimated the survival rates for 3 and 5 years.

## DISCUSSION

Establishing a standardized clinical classification agreed to by most experts is important for the general survey, diagnosis, treatment, and prognosis evaluation of advanced schistosomiasis. Because of the complex complications of advanced schistosomiasis, experts had different opinions on classification for more than half a century, until the novel classification was proposed in 2012.^4^ The complications of advanced schistosomiasis are interrelated. Thus, network analysis was used to explore the interrelationships of the isolated complications of advanced schistosomiasis. These 15 isolated complications covered most of the previous sub-types and include novel sub-types reported in recent years.^9^^,^^10^^,^^15^ These isolated complications were further clustered into three major categorizes (clustered complications). A nomogram based on the clustered complications provided an individualized tool for guiding follow-up and helping prognosis evaluation in advanced schistosomiasis.

### Schistosomal abnormal metabolic syndromes.

Six isolated complications were identified in SAMs. None of them had ever been mentioned in previous classifications. However, these co-occurring complications were not uncommon in advanced schistosomiasis. A median survival rate of SAMs was found among the three clusters. The central nodes of SAMs were CKD and pulmonary hypertension, also known as schistosomiasis-associated pulmonary arterial hypertension (Sch-PAH).

CKD could be caused by various reasons, including chronic renal structure and dysfunction. Immune complex-mediated kidney disease was related to host immune responses to the parasite antigens.^16^ A recent study^17^ showed that several types of glomerulopathy, including membranous nephropathy, were observed in patients with schistosomiasis. As was shown in the network that CKD was very close to the sub-net of SIGs (caused by inflammatory granulomatous). Our data showed that the incidence rate of advanced schistosomiasis complicated by metabolic syndrome was very high. Metabolic syndrome could be both a risk factor and a consequence of CKD.^18^^,^^19^ Furthermore, metabolic syndrome was associated with the presence of clinically significant fibrosis and hemodynamics changes.^20^^,^^21^

Glomerulopathy, portal hypertension, and chronic renal anemia may play roles in Sch-PAH.^22^ Previous studies showed that Sch-PAH survival was significantly better than that of idiopathic pulmonary hypertension.^23^ COPD, respiratory failure, and right heart failure were more likely the consequence of Sch-PAH,^24^ as a strong intercorrelation was observed between these nodes.

### Schistosomal abnormal hemodynamics syndromes.

Four isolated complications were identified in SAHs. They overlapped with the sub-type of hemorrhage of Deng et al.^4^ (gastroesophageal variceal bleeding and gastrointestinal ulcer bleeding) and hepatic coma (hepatic encephalopathy). The central nodes of SAHs were gastroesophageal variceal bleeding and gastrointestinal ulcer bleeding.

Gastroesophageal variceal bleeding was the major cause of the low survival rate in advanced schistosomiasis. Patients who experienced one episode of gastroesophageal variceal bleeding were likely to experience more than one and would die from uncontrolled bleeding.^25^ Only one study^26^ reported a case of recurrent bleeding by gastrointestinal ulcer bleeding, and it did not respond to anti-ulcer therapy or endoscopic hemostasis methods. Gastrointestinal hemorrhage, including both gastroesophageal variceal bleeding and gastrointestinal ulcer bleeding, might lead to hepatic encephalopathy in advanced schistosomiasis with liver dysfunction. However, gastrointestinal ulcer bleeding is more relevant to hepatic encephalopathy than minimal hepatic encephalopathy. Our results indicated that gastrointestinal ulcer bleeding might play a large role in engendering minimal hepatic encephalopathy/hepatic encephalopathy. However, recessive gastrointestinal ulcer bleeding was usually neglected in advanced schistosomiasis.

Hepatic encephalopathy, a central nervous system metabolic disorder syndrome, was another main cause of the low survival rate in advanced schistosomiasis. As the mild form of hepatic encephalopathy, minimal hepatic encephalopathy is usually neglected clinically, and requires specific diagnostic methods.^11^ Interestingly, our network showed that hepatic encephalopathy was closer to SIGs, whereas minimal hepatic encephalopathy was closer to SAMs. Our results suggested that hepatic encephalopathy might be a consequence triggered by complications derived from portal hypertension, but minimal hepatic encephalopathy might be a persistent, mild metabolic abnormality.^27^

An underestimate was shown by the nomogram in predicting 3- and 5-year survival rates of advanced schistosomiasis. The main reason might be that most of the patients with SAHs, with gastroesophageal variceal bleeding and hepatic encephalopathy, die within 1 year after their first visit to the hospital, but those with minimal hepatic encephalopathy might last several years without obvious progression.

### Schistosomal inflammatory granulomatous syndromes.

Five isolated complications were identified in SIGs. For the most part, all were covered by the classification of Deng et al.^4^ The survival rate was high in SIGs, and the central nodes of SIGs were fibrosis and ascites.

Fibrosis, ascites, and splenomegaly are results of typical presinusoidal portal hypertension secondary to hepatic symmetric pipestem fibrosis led by inflammatory granulomas.^28^ The mildly to moderately enlarged liver may slowly progress for decades, which is induced by long-term or repeated action of one or more causes (usually hepatitis C virus or hepatitis B virus, or associated nonalcoholic steatohepatitis).^9^

Colonic proliferation (including colorectal polyp and colorectal cancer) is led by egg deposition in the colorectal mucosa, which leads to inflammatory granuloma formation, congestion, edematous polyp formation, and ulceration.^9^ Colorectal polyp is generally considered the premalignant precursor from which colorectal cancer arises.^29^ A low survival rate in colonic proliferation was observed, which might be caused by the low incidence of colorectal cancer brought about by the Shanghai colorectal cancer screening program launched in 2012.^30^

Our study had some limitations. First, *S. japonicum* is mainly prevalent in China, and the clustered complications of SAMs, SAHs, and SIGs may not be suitable for other schistosomal species. Second, because of the monocentric and retrospective nature of our study, it might have selection bias. The epidemiology we reported was regional, which might not be in line with the characteristics of the disease nationwide. Personal lifestyle and eating habits might be related to the occurrence of diseases, and these variables were not included in the influencing factors. Third, cholecystitis, cholangitis, appendicitis, and urinary tract infection were also commonly noticed in advanced schistosomiasis. However, because of the lack of reports in the literature on the primary targets of these locations in *S. japonicum*, we did not include them in the analysis. More comprehensive research is needed.

In conclusion, network analysis identified three major clustered complications in advanced schistosomiasis—SAHs, SAMs, and SIGs—with a low, median, and high survival rate, respectively. A nomogram based on the clustered complications was useful in predicting the 1-year survival rate of advanced schistosomiasis.

## Supplemental files


Supplemental materials
