# Supplementary material for: Network Analysis and Nomogram in the Novel Classification and Prognosis Prediction of Advanced Schistosomiasis Japonica
Source: Am J Trop Med Hyg. 2023 Jan 23;108(3):569–77. doi: 10.4269/ajtmh.22-0267 (PMC9978554; doi:10.4269/ajtmh.22-0267)
Supplement: Supplementary file 1 [file tpmd220267.SD1.pdf]

Supplementary Table 1. Node centrality of the training cohort and the validation cohort

Nodes	Measure	Training cohort (Z-score)	Validation cohort (Z-score)
Metabolic syndromes	Betweenness	0.47	0.51
Hepatic encephalopathy	Betweenness	-0.83	-0.83
Minimal hepatic encephalopathy	Betweenness	-0.83	-0.83
Chronic obstructive pulmonary disease	Betweenness	0.13	-0.09
Respiratory failure	Betweenness	-0.83	-0.83
Pulmonary hypertension	Betweenness	-0.74	-0.76
Right heart failure	Betweenness	-0.65	-0.83
Gastroesophageal variceal bleeding	Betweenness	-0.31	0.85
Gastrointestinal ulcer bleeding	Betweenness	1.17	1.99
Splenomegaly	Betweenness	0.04	-0.29
Fibrosis	Betweenness	0.13	0.78
Chronic kidney disease	Betweenness	2.64	1.92
Ascites	Betweenness	1.08	-0.83
Colorectal polyp	Betweenness	-0.74	0.04
Colorectal cancer	Betweenness	-0.74	-0.83
Metabolic syndromes	Closeness	0.55	0.82
Hepatic encephalopathy	Closeness	-0.92	-2.09
Minimal hepatic encephalopathy	Closeness	-0.99	-0.38
Chronic obstructive pulmonary disease	Closeness	0.01	0.16
Respiratory failure	Closeness	-0.73	-0.12
Pulmonary hypertension	Closeness	-0.70	0.35
Right heart failure	Closeness	0.15	0.40
Gastroesophageal variceal bleeding	Closeness	0.28	1.04
Gastrointestinal ulcer bleeding	Closeness	0.67	1.25
Splenomegaly	Closeness	0.92	-0.57
Fibrosis	Closeness	0.63	1.26
Chronic kidney disease	Closeness	1.39	0.90
Ascites	Closeness	1.67	-0.99
Colorectal polyp	Closeness	-1.61	-0.82
Colorectal cancer	Closeness	-1.33	-1.22
Metabolic syndromes	Strength	-0.40	-0.29
Hepatic encephalopathy	Strength	-1.24	-1.40
Minimal hepatic encephalopathy	Strength	-1.10	-1.19
Chronic obstructive pulmonary disease	Strength	0.61	0.78
Respiratory failure	Strength	-0.23	0.54
Pulmonary hypertension$	Strength	0.71	0.88
Right heart failure	Strength	-0.27	0.44
Gastroesophageal variceal bleeding*	Strength	0.80	0.48
Gastrointestinal ulcer bleeding*	Strength	1.60	1.31
Splenomegaly	Strength	0.06	-0.60

Fibrosis#	Strength	0.12	-0.29
Chronic kidney disease$	Strength	1.29	1.99
Ascites#	Strength	0.96	-0.87
Colorectal polyp	Strength	-1.42	-0.82
Colorectal cancer	Strength	-1.50	-0.96

$, *, #, the most important nodes in the sub-net of Schistosomial abnormal metabolic syndromes (SAMs), Schistosomial abnormal hemodynamics syndromes (SAHs), and Schistosomial inflammatory granulomatous syndromes (SIGs), respectively.

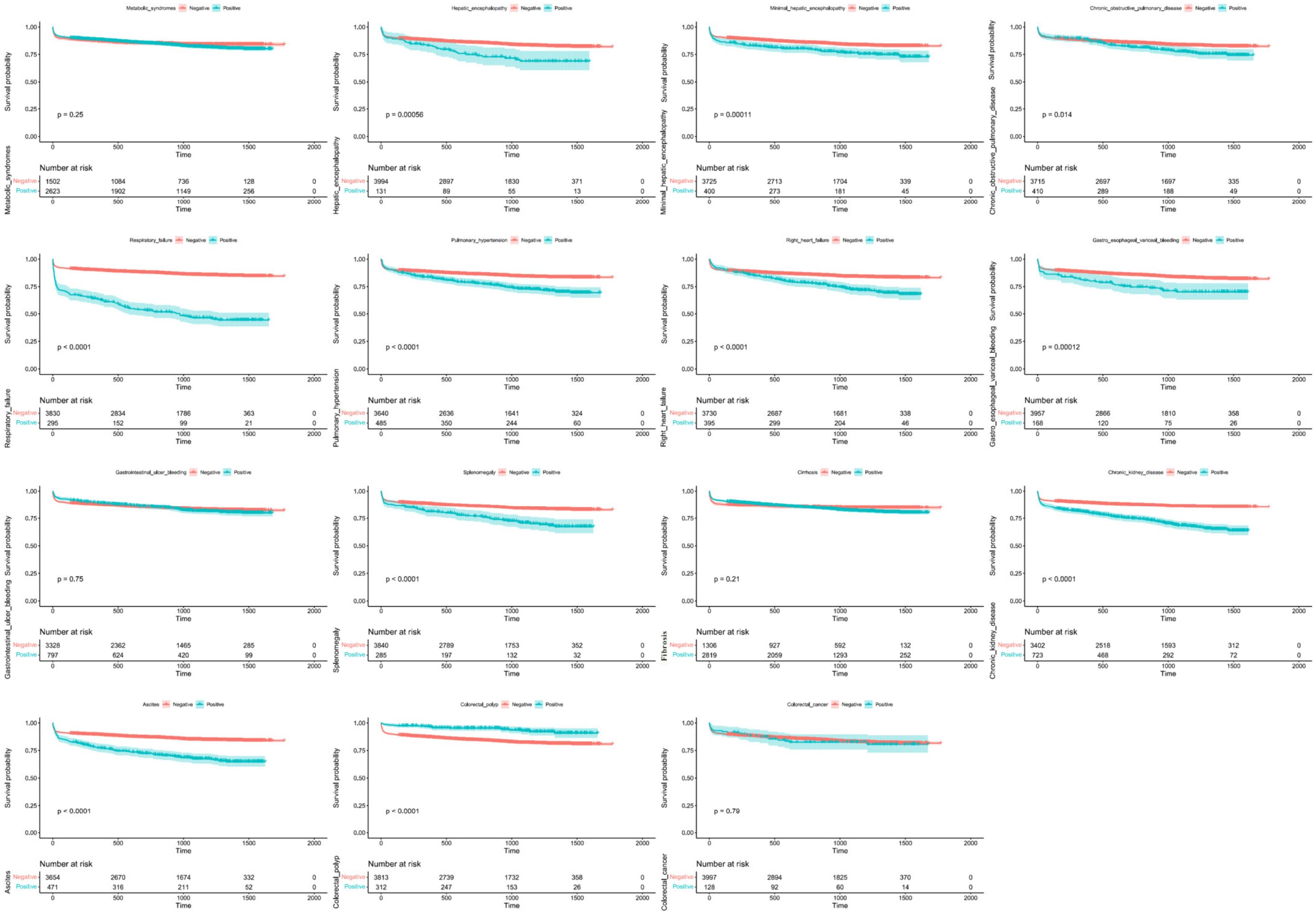

Supplementary Fig 1. Five-year survival rate of the isolated complications.

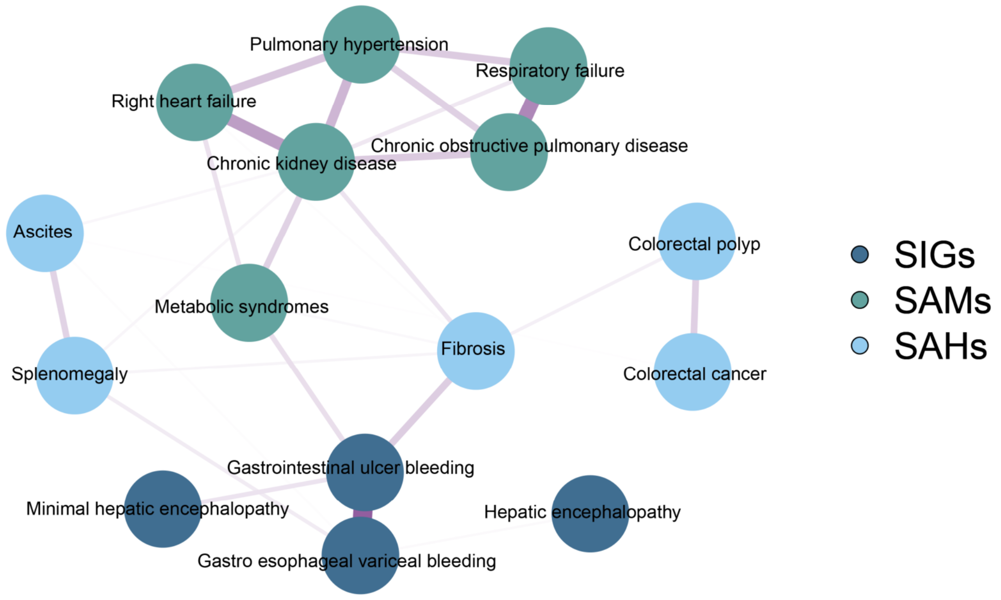

Supplementary Fig 2. Similar network structure (with that in the training cohort) and three major clusters are seen in the validation cohort.

SAHs, Schistosomial abnormal hemodynamics syndromes; SAMs, Schistosomial abnormal metabolic syndromes; SIGs, Schistosomial inflammatory granulomatous syndromes.
